# Use of tiling array data and RNA secondary structure predictions to identify noncoding RNA genes

**DOI:** 10.1186/1471-2164-8-244

**Published:** 2007-07-23

**Authors:** Christian Weile, Paul P Gardner, Mads M Hedegaard, Jeppe Vinther

**Affiliations:** 1 Molecular Evolution Group, Department of Molecular Biology, University of Copenhagen, Ole Maaløes Vej 5, Building 4.1.27, DK-2200 Copenhagen N, Denmark

## Abstract

**Background:**

Within the last decade a large number of noncoding RNA genes have been identified, but this may only be the tip of the iceberg. Using comparative genomics a large number of sequences that have signals concordant with conserved RNA secondary structures have been discovered in the human genome. Moreover, genome wide transcription profiling with tiling arrays indicate that the majority of the genome is transcribed.

**Results:**

We have combined tiling array data with genome wide structural RNA predictions to search for novel noncoding and structural RNA genes that are expressed in the human neuroblastoma cell line SK-N-AS. Using this strategy, we identify thousands of human candidate RNA genes. To further verify the expression of these genes, we focused on candidate genes that had a stable hairpin structures or a high level of covariance. Using northern blotting, we verify the expression of 2 out of 3 of the hairpin structures and 3 out of 9 high covariance structures in SK-N-AS cells.

**Conclusion:**

Our results demonstrate that many human noncoding, structured and conserved RNA genes remain to be discovered and that tissue specific tiling array data can be used in combination with computational predictions of sequences encoding structural RNAs to improve the search for such genes.

## Background

The sequencing of the human genome marked the starting point of a very difficult task: to make sense of the enormous amount of information stored in the genome by annotating the functionally important regions. Emphasis was initially put on the protein coding DNA sequences, which are generally well conserved and can easily be converted into the corresponding protein sequence. However, in recent years it has become clear that large parts of the noncoding DNA present in the human genome is functional and that noncoding genes may be as abundant as protein coding genes [1].

Central to this realization has been the sequencing of additional mammalian genomes. Comparative genomics have demonstrated that the fraction of the human genome that is under purifying selection is much larger than the part that makes up the protein coding sequence, suggesting that many non protein coding regions of the genome have important functions [2]. Conserved sequence elements in promoter, intron and untranslated regions (UTRs) control transcription and processing of mRNAs [3]. Moreover, distant enhancer elements also influence transcription over long distances.

In fact, such noncoding enhancer elements are the most highly conserved regions of the human genome [4]. Another class of conserved noncoding sequence is the RNA genes that are transcribed, but does not encode any protein. Instead the functions of these genes depend on the RNA itself, which can be unstructured or adopt functional secondary structures through internal base pairing or pairing to other RNA molecules.

In this way RNA can act as enzymes, structural scaffolds and cofactors for proteins. Structural RNA gene sequences are often less well conserved than protein coding and regulatory sequences, since it is the RNA secondary structure that is conserved rather than the primary sequence. Recently, computational methods that can detect the signatures of conserved RNA structure in aligned DNA sequences have been developed and have revealed that the human genome contains many thousands of potential structural RNA genes [5,6]. Some of these can be assigned to known RNA gene families such as tRNA, rRNAs, snoRNAs and miRNAs, while others have no assigned functions. A common theme seems to be that many noncoding RNA genes have a very restricted expression. Often, they have low or no EST coverage, but this does not necessarily mean that they are not expressed and nonfunctional [7]. An interesting example of this is the noncoding RNA (ncRNA) HAR1F that has undergone strong positive selection in the human lineage and are expressed only in Cajal-Retzius neurons in the developing human neocortex from 7 to 19 gestational weeks [8]. Such spatial and temporal restricted expression makes it a daunting task to verify expression of computationally predicted structural RNAs [9]. This may be especially true for RNA genes expressed in the brain, which is a very complex organ estimated to have thousands of different cell types.

Advances in array technology have allowed unbiased genome wide analysis of RNA transcription using tiling arrays of overlapping probes spanning the entire euchromatic part of the human genome [10,11]. These RNA expression studies demonstrate that a large proportion of the human genome is transcribed and that the transcription is more complex than previously anticipated with extensive use of alternative promoters, splicing and polyadenylation. So far tiling array analysis has been performed on RNA from a limited number of cell lines, but these experiments nevertheless indicate that large parts of the human genome are transcribed. These findings are supported by findings from large scale cDNA cloning efforts that also find high transcriptional diversity and many ncRNAs [12].

We have combined data from structural RNA gene prediction [9] with tiling array data from the neuroblastoma cell line SK-N-AS [10,13] to identify novel structural RNA genes expressed in this cell line. Using this strategy, we identify thousands of human candidate RNA genes that are most likely expressed in SK-N-AS cells. The list of candidates can be found at the CRUFTS homepage [14]. For verification of expression we focused on candidates having energetically favorable hairpin structures or a high level of covariance. Using northern blotting, we verify the expression of 2 out of 3 of the hairpins structures. Moreover, 3 out of 9 of the structures with high covariance could be detected by northern in SK-N-AS cells.

## Results and discussion

The identification of ncRNAs has been facilitated by comparative genomics and development of methods to detect RNA expression on a genome wide scale. In this work we combine genome tiling array expression data [10,13] with genome sequence conservation [2] and secondary structure information [15] in an effort to identify novel ncRNAs in the human genome.

The genome tiling array data is derived from phase 2 of Affymetrix tiling array studies [10]. Here, 10 chromosomes (6, 7, 13, 14, 19, 20, 21, 22, X and Y) of the human genome, corresponding to ~30% of the non-repetitive portion of the genome, are tiled upon microarrays at 5 base-pair intervals. Only non-repetitive regions are tiled due to the risk of cross hybridisation and the difficulty of determining which genomic region a multi-copy transcript is derived from. For this study we have used data from the neuroblastoma cell line (SK-N-AS) that was analyzed using a hidden Markov model trained to discriminate between transcribed and untranscribed regions [13]. The combined conservation and secondary structure track is derived from a study using structural information on the conserved fraction of the human genome [2,16]. The method is based upon a secondary structure prediction algorithm for folding sequence alignments [17] combined with an algorithm (called RNAz) [9] that has been trained to discriminate between sequence alignments of ncRNA sequences and their randomized counterparts [9].

**Figure 1:**
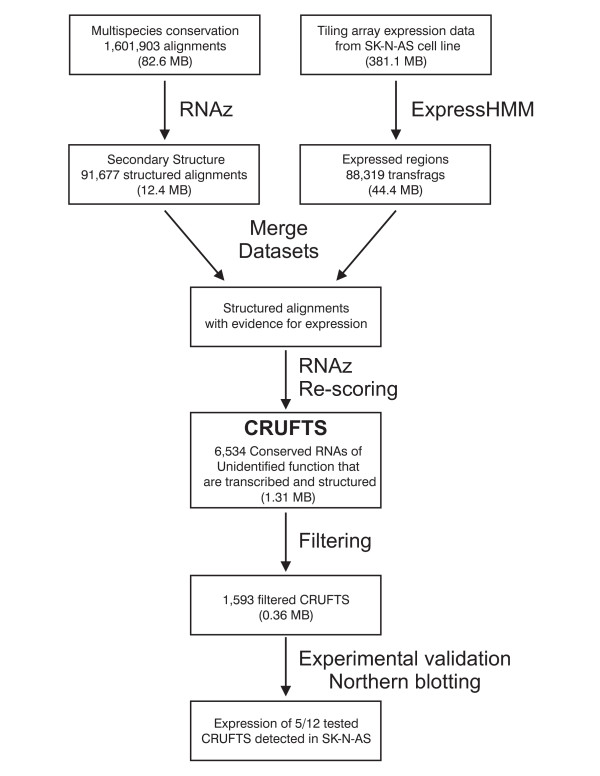
Strategy used to identify structural non coding RNA genes Schematic representation of the work-flow used to identify and verify CRUFTS. Multispecies conservation data [2], structured alignments [43] and the tiling array data [10,13] have all been published. For details and references see main text.

We intersected 88,319 genomic regions predicted to be expressed in SK-N-AS cells by tiling array analysis [10,13] with 91,677 genomic regions predicted to contain conserved secondary structure (Figure 1)[18]. To improve sensitivity, we used the least conservative prediction of secondary structure for the intersection. To further improve the predictions, we obtained multi-species alignments from UCSC table browser [19] of human (hg17), chimpanzee (panTro1), dog (canFam1), mouse (mm5), rat (rn3), chicken (galGal2), zebrafish (danRer1) and Fugu (fr1) for the regions that showed evidence of both expression and structure. These alignments were re-scored with RNAz using more stringent settings. This produced 32,439 CRUFTS (Conserved RNAs of Unidentified Function that are Transcribed and Structured), which when collapsed into overlapping regions these map to 6,534 unique genomic regions.

**Figure 2:**
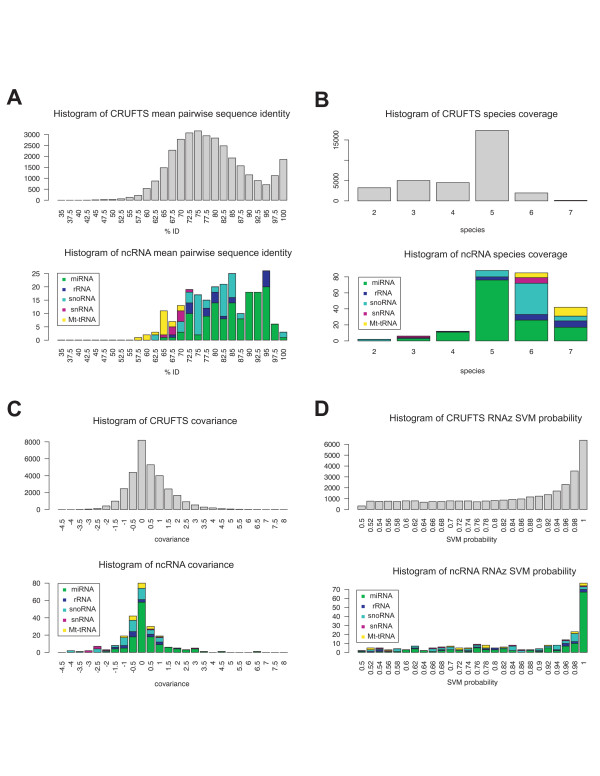
Characteristics of CRUFTS and known ncRNAs. Histograms showing the distributions of mean-pairwise sequence identity A), species-coverage B), covariance C) and RNAz probability D) for the CRUFTs and known ncRNAs.

To investigate if the CRUFTS contained already known ncRNAs, we used available annotations of human ncRNAs [20-24]. The ncRNAs used were: Xist, Telomerase RNA, HVG-1,2&3, H19, RNase MRP, RNase P, tRNAs, Pseudo-tRNAs, rRNAs, small cytosolic RNAs (SRP, hY1, hY3, hY4, hY5), miRNAs and snoRNAs. The classical ncRNAs such as rRNA, tRNA, SRP etc. are classified as repeats by RepeatMasker [25] and are therefore not present in the CRUFT dataset. Also, some rRNA, tRNAs and SRPs were absent in the final set due to difficulties of producing correct genome alignments for these regions, which is critical for secondary structure prediction with RNAz. In subsequent versions of the genome alignments (17-way and beyond) these difficulties appear to have been overcome [26,27]. Of the 32,439 CRUFTS, 240 overlap the remaining known ncRNAs in our control data set (see Table 1), consistent with not all of these being expressed in the SK-N-AS cell line and not all ncRNA being detected by the RNAz algorithm. Moreover, it is noteworthy that the SK-N-AS tiling array data used for our analysis is based on hybridization of cDNA originating from polyA selected RNA to the array, which probably excludes some ncRNAs from the CRUFTS dataset. All in all, after removing the known ncRNAs and CRUFTS overlapping 3' UTRs, we have 5,629 potential novel non-overlapping ncRNAs in the CRUFTS dataset. To further refine the dataset and reduce the number of false positive among the CRUFTS, we compared a number of parameters for the CRUFTS with those from the known ncRNAs (Figure 2). We find that the CRUFTS have a mean pairwise identity (PID) distribution that is similar to that of the control ncRNA set, except that many more CRUFTS have structures that have PIDs above 95% (Figure 2A). Previously, it has been shown that secondary structure signals are largely lost below 65% identity and above 95% identity there is little supporting information from mutational analysis [28]. Moreover, the RNAz algorithm detects many structures having PID above 95% and it is currently not known, if these represent new structural RNAs that are more highly conserved than known ncRNAs or false positives [29]. We also noted that that CRUFTS generally have sequence coverage in fewer species than the known ncRNAs (Figure 2B), which reflects that the ncRNAs in the known ncRNA set are well conserved. The covariance and RNAz SVM probability distributions of the CRUFTS are similar to the corresponding distributions of the ncRNAs (Figure 2C and 2D), but the known ncRNAs cluster in the RNAz high probability fraction. After considering the distributions of these different statistics, we applied the filters shown in Table 2 to enrich for CRUFTS resembling the known ncRNAs in the dataset. These filters resulted in a 10-fold reduction of the amount of data (from 32439 to 3243 CRUFTS or 6534 to 1593 non-overlapping regions) and increased the enrichment of known ncRNAs 2.17 fold, which is highly significant (p = 6.6e-8) (see Table 1). Of the 1593 non-overlapping regions present in the filtered CRUFTS dataset, 1314 are potential novel ncRNAs (i.e. not a known ncRNA and not located in an 3 ' UTR).

**Table 1:** Enrichment of known ncRNAs in subsets of CRUFTS

Scheme/Overlap	ncRNA Enrichment	ncRNA Families
All	1.00 (1.00)	135 miRNA, 21 rRNA, 58 snoRNA, 9 snRNA, 17 Mt-tRNA
Filtered (Table 2 parameters)	2.17 (6.630e-08)	miRNA, rRNA, snoRNA, snRNA, Mt-tRNA

mRNA/EST	0.64 (1.000)	miRNA, snRNA, snoRNA
mRNA/EST (no UTR or exon)	2.00 (7.000e-05)	miRNA, snRNA, snoRNA
5' UTR	0.97 (0.5806)	miRNA
Intron	0.97 (0.1148)	miRNA, rRNA, snoRNA, Mt-tRNA
3' UTR	0.00 (1.000)	-
Intergenic	1.21 (4.448e-03)	miRNA, rRNA, snRNA, snoRNA

EvoFold	4.67 (5.271e-06)	miRNA
InDel selection	1.74 (<2.2e-16)	miRNA, rRNA, snoRNA
InDel selection (no miRNA)	0.46 (0.4115)	rRNA, snoRNA
Transposonfree (10 k)	0.51 (0.9786)	miRNA
Transposonfree (5 k)	1.17 (0.09059)	miRNA, rRNA, snoRNA

Covariance top 300	4.05 (4.277e-04)	miRNA, snoRNA, snRNA
RNAz probability top 300	6.76 (8.367e-09)	miRNA

Enrichment values of known ncRNAs in each filtering method or genome annotation. Column 2 contains the degree of enrichment of the known ncRNAs for each dataset compared to the "All" CRUFTS dataset. P-values for the enrichment were calculated using Fishers' exact test. In the final column, the ncRNA families contained within the annotation is indicated. See text for details and references.

**Table 2:** Parameters used for filtering the CRUFTS

Feature	Threshold
RNAalifold covariation measure	< 0
Number of species	>4
Mean pairwise sequence identity	65% < and < 95%
RNAz SVM probability	> 0.90

To further characterize our CRUFTS data set we mapped a number of other genome annotations to the CRUFTS. Using annotations from the Refseq database [30] and the human EST database, we find that CRUFTS overlapping with known ncRNA are enriched in intergenic regions and regions that have mRNA/EST evidence, but no overlapping exon or a UTR sequence (see Table 1). This corresponds to what one would expect given the types of ncRNAs in the control ncRNA set and suggests that CRUFTS located in intergenic regions and having mRNA/EST evidence, but no overlapping exon or a UTR sequence are more likely to represent true ncRNA genes.

Of particular interest is a study by Pedersen et al. that implemented a probabilistic approach (called EvoFold) based on phylogenetic stochastic context-free grammars to predict conserved secondary structures in the human genome [6]. In contrast to the RNAz algorithm, EvoFold does not use folding energy to predict RNA structures, but rather calculates the probability of an RNA structure, while taking the phylogeny into consideration. We find that the EvoFold and RNAz CRUFTS enrich for known miRNAs (Table 1, p = 5.2e-6), showing that these two structural RNA gene finders complement each other and that the CRUFTS overlapping EvoFold predictions are more likely to be miRNAs than the CRUFTS in general. Many of the CRUFTS are located in intergenic regions that have no known function. Two approaches that have the potential to detect genomic regions that are under purifying selection have recently been published [31,32]. Lunter et al. searched the genome for insertion and deletion (indel) free regions [31] and found clear evidence of purifying selection against indels in many regions of the genome. Interestingly, the majority of indel free regions are located outside protein coding genes and most known miRNA genes are located within indel free regions [31]. We find that CRUFTS that overlap an indel free region of the genome are significantly enriched in known ncRNA (Table 1, P-value < 2e-16). These observations suggest that the CRUFTS that overlap indel free regions of the human genome are more likely to be ncRNAs (and miRNAs in particular) that have important functions sensitive to insertions and deletions in the sequence. Simons et al. have made a similar analysis of transposon-free regions of the human genome [32]. As shown in Table 1 the CRUFTS overlapping transposon-free regions were only slightly enriched for ncRNAs (P = 0.09 for the 5 kb regions), indicating that the known ncRNA are rather insensitive to insertion of transposons in a 5 kb window containing the ncRNA. All the CRUFT datasets and the annotation of these can be accessed at the CRUFTS homepage [14].

**Figure 3:**
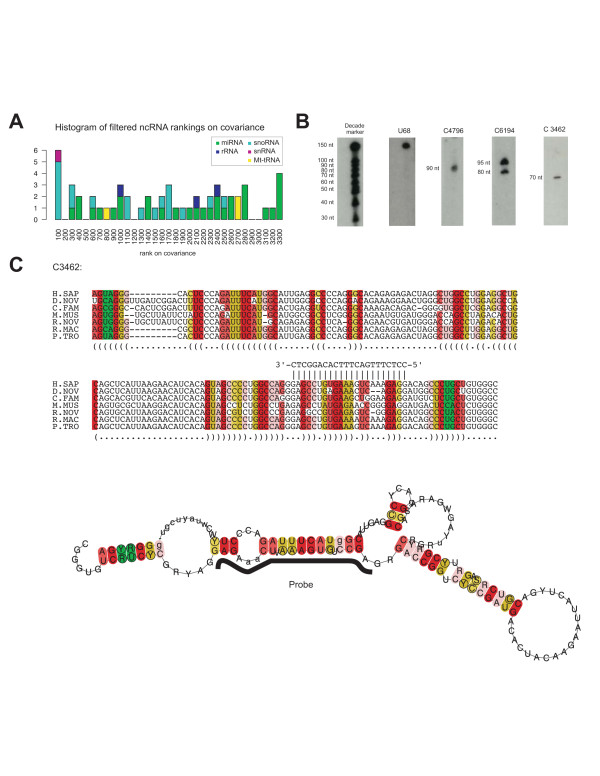
Experimental verification of CRUFTS showing high RNAz covariance. A) Histogram showing enrichment of known ncRNAs in the top 300 CRUFTS sorted on covariance. B) Northern blots with specific LNA modified DNA probes for three high covariance CRUFTS. The U68 snoRNA was used as positive control. C) Alignment and conserved secondary structure of the CRUFTS C3462. The location of the probe used for detection is indicated. The positions in the alignments and the secondary structure are color-coded according to the conservation of the basepair interaction following the RNAz conventions [9]. Green indicates that 3 different types of pairs (e.g. G-C in human, G-U in dog and A-U in zebrafish) support the interaction. Yellow color coding indicates that the base pair is supported by 2 types of pairs and red that only a single pair-type supports the interaction. The intensity of color coding is fated with the number of sequences in conflict with the predicted interaction.

Next, we wanted to experimentally verify the expression of some of the CRUFTS in the SK-N-AS cell line. When CRUFTS were ranked on the RNAalifold measure of covariance [17] known ncRNAs including miRNAs, snoRNAs and snRNAs were enriched in the top 300 rankings (p = 4.3e-4)(see Table 1 and Figure 3A). We choose 9 structures from the top 25 CRUFTS ranked on covariance and designed complementary probes for northern blotting. Using RNA enriched for small RNAs and isolated from SK-N-AS cells, three out of the nine selected CRUFTS could be repeatedly detected by northern blotting using LNA modified DNA probes (Figure 3B). As a positive control we used the U68 H/ACA snoRNA, which ranked high on the covariance sorted list. A list of these investigated CRUFTS along with their predicted structures and the probes sequences can be found in Additional file 1 and is exemplified for C3462 in figure 3C. The CRUFTS that were not detected by our northern blots may represent sequences that are not RNA genes or RNA genes that are expressed in SK-N-AS cells at levels below the detection level of our northern blots.

Alternatively, they may be expressed as part of long RNA transcripts that would not be detected in our northerns or be processed into smaller RNAs not targeted by our probes. The three CRUFTS that are detected by our probes do not match any of the profiles in the RFAM database and do not resemble any previously described ncRNA gene. The probes hybridize to RNAs in the range between 70 and 95 bp. This size range is typical of C/D snoRNAs [23], but none of the candidates have canonical CD boxes, indicating that these CRUFTS expressed in the SK-N-AS cell line are not snoRNAs, but belong to currently uncharacterized ncRNA genes families. The C4796 CRUFTS is located intergenic, whereas C6194 and C3462 are located introns of latent transforming growth factor beta binding protein 2 (LTBP2) and transmembrane protease, serine 6 (TMPRSS6), respectively. All the three detected covariance CRUFTS are located in indel free regions [31]. UCSC screenshots of the genomic neighborhoods of the detected covariance CRUFTS can be found in Additional file 2.

**Figure 4:**
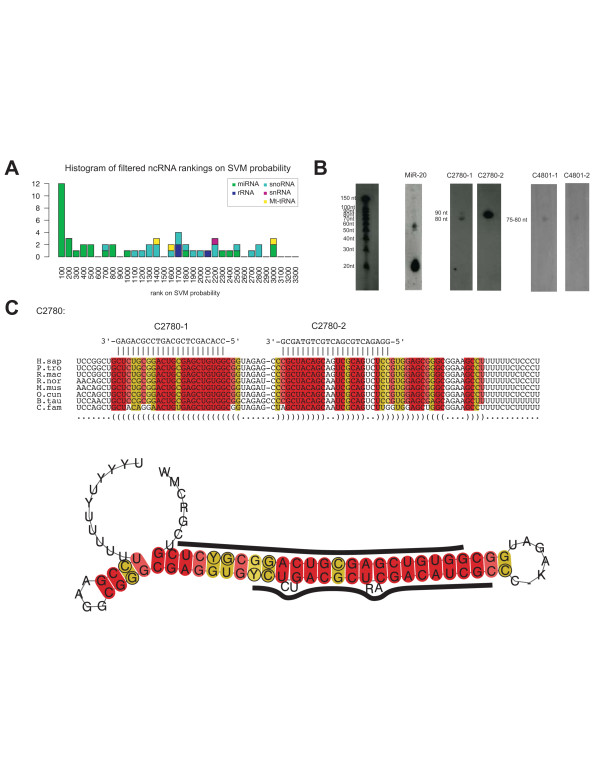
Experimental verification of CRUFTS showing high RNAz SVM probability. A) Histogram showing enrichment of known ncRNAs in the top 300 CRUFTS sorted on RNAz SVM probability B) Northern blots with specific LNA modified DNA probes for three high covariance CRUFTS. The U68 snoRNA is the positive control. C) Alignment and conserved secondary structure of the CRUFTS C3462. The location of the probe used for detection is indicated. The positions in the alignments and the secondary structure are color-coded according to the conservation of the basepair interaction following the RNAz conventions [9]. Green indicates that 3 different types of pairs (e.g. G-C in human, G-U in dog and A-U in zebrafish) support the interaction. Yellow color coding indicates that the base pair is supported by 2 types of pairs and red that only a single pair-type supports the interaction. The intensity of color coding is faded fates with the number of sequences in conflict with the predicted interaction.

The RNAz algorithm is dependent on folding energy and since miRNA genes generally form stable secondary structures consisting of a hairpin, RNAz shows high sensitivity for miRNA genes [9,33,34]. When the CRUFTS were sorted according to their RNAz SVM probability, known and predicted miRNA genes were enriched in the top 300 ranking (p = 8.4e-9, see Figure 4A, Table 1) and many structures with miRNA like hairpins can be observed. We found that three of the CRUFTS within the TOP 300 RNAz SVM rankings overlapped with miRNAs candidates that previously have been predicted by phylogenetic shadowing by Plasterk and coworkers [35] and also the indel free regions described by Lunter et al. [31]. Using LNA modified DNA probes complementary to each side of these hairpin structures (2 probes for each candidate structure, see Figure 4C and Additional file 1), two of the three probe pairs hybridized specifically to SK-N-AS RNA enriched for small RNAs (Figure 4B).

However, the signals observed with these probes were all in the 75–90 nt. range and we see no signal in the size range of mature miRNA. This was not due to loss of small RNAs in our RNA preparation, since a known miRNA (miR-20) was detected with a miR-20 specific probe (Figure 4A). The fact that we observe a signal of similar size with probes targeted to both sides of the putative miRNA hairpins indicates that the probes do detect a pre-miRNA like RNA hairpin expressed in the SK-N-AS cell line.

During the course of this study, expression of the mature form of CRUFTS C4801 (candidate 225 from Berezikov et al., [35]) has been verified by cloning from mouse brain and by a modified microarray-based detection system (RAKE) [36]. Previously, it has been observed that miRNA-138 accumulates in the pre-miRNA form in the cytoplasm in some tissues and are only processed to the mature form in restricted tissues [37]. We have tested a panel of cell lines originating from different tissues with probes for C2780 and C4801 and find that 75–90 nt RNAs are detected in most cell lines and tissues, but no RNAs corresponding to mature forms (~21 nts.) (Additional file 4). It is therefore possible that miRNA processing of C4801 and possibly C2780 is regulated and occurs only in restricted tissues. However, we cannot completely rule out that we fail to detect the mature miRNA forms of these CRUFTS miRNA candidates because our northern probes do not have sufficient overlap with the mature form of the miRNA. UCSC screenshots of the genomic neighborhoods of the detected hairpin CRUFTS can be found in Additional file 3 online. Interestingly, C4801 is located close to miR-99b, miR-125a and miR-let-7e on chromosome 19, suggesting that C4801 is a new member of this miRNA cluster.

Other studies have used strategies that a similar to ours in order to identify novel ncRNAs. Babak et al. [38] used the QRNA algorithm [39] to search for ncRNAs in human-mouse pairwise alignments from intergenic and intronic regions conserved between human and mouse and rat. A custom mouse DNA array with 6 probes for each of 3,478 predicted ncRNAs was hybridized with RNA from 16 mouse tissues.

The 55 candidates that showed the highest signal on the array were chosen for northern blotting, which confirmed the expression of 8 candidates. Surprisingly, none of these candidates could be detected in human tissues, leading the authors to speculate that conserved and transcribed intergenic and intronic regions are not independent functional elements, but may have species or lineage specific functions [38]. Babak et al. also investigate the overlap between their candidates and tiling array data [10] and find that they do not overlap more than what would be expected by chance. Our study is not directly comparable with the study of Babak et al. We have used multiple alignments and RNAz [9] rather than pairwise alignments and QRNA [39] to predict conserved secondary structure. Moreover, we use the properties of the predicted secondary structures and the tiling data for filtering our predictions before verifying expression by northern blotting. These differences may explain that we have a higher success rate in our northern verifications. In another study, Washietl et al. used RNAz [9] and EvoFold [6] secondary structure predictions to identify potential ncRNAs in the ENCODE regions [29]. From a selection of 175 high-scoring predictions that was aided by visual inspection, 43 were detected by RT-PCR on RNA isolated from 6 different tissues. Interestingly, the predictions that are supported by tiling array expression were more likely to yield positive RT-PCR results (29% compared to 19% without support from tiling) [29]. These results support our finding that is possible to enrich for structural RNA genes by combining RNA structure predictions with tiling array data.

## Conclusion

We have integrated tiling array expression data with different annotations derived from comparative genomics to search for structural RNA genes that are expressed in the human neuroblastoma cell line SK-N-AS. In this way, we identified several thousand genomic regions (CRUFTS) that are strong candidates for being structural RNA genes. Using northern blotting, we verified the expression of 5 out of 12 investigated CRUFTS in the SK-N-AS cell line. Three of the verified CRUFTS can not be assigned to existing ncRNA families and could belong to novel ncRNA classes. The remaining two CRUFTS, which were detected by northern blotting, probably belong to the miRNA family. Our results indicate that many human noncoding, structured and conserved RNA genes remain to be discovered and that tiling array data can be used in combination with computational predictions of structural RNAs to detect novel ncRNA genes. Our strategy could easily be applied to other tiling array datasets and new annotations from comparative sequence analysis and should facilitate the identification of novel ncRNAs. The CRUFTS data can be accessed at the CRUFTS homepage [14].

## Methods

### Bioinformatic analysis

To produce a set of predictions enriched for both novel and known ncRNAs, we located overlapping regions of a conserved, structured RNA-like and an unbiased transcription annotation. The essential features of our pipeline are outlined in Figure 1. Beginning with the 88,319 genomic regions from the least conservative mammalian RNAz annotation [9,40] and 93917 genomic regions from the ExpressHMM analysis of Affymetrix phase 2 human genome tiling arrays [13,41], we produced a dataset using the UCSC table browser [19] of 4,160 genomic regions that overlapped both the RNAz and expressHMM predictions.

From these regions 7,703 alignments of genomic regions from human (hg17), chimpanzee (panTro1), dog (canFam1), mouse (mm5), rat (rn3), chicken (galGal2), zebrafish (danRer1) and Fugu (fr1) within the resulting regions were obtained using the UCSC table browser. These alignments were fed into the RNAz algorithm and rescored using the following parameters. The alignments were sliced into 120 long blocks with a step size of 20 and only alignments with more than 65 columns were reported. All slices with an SVM derived probability greater than 0.5 were reported. Both strands of the genome were tested for structure potential as the tiling array data is not strand specific. This resulted in 32,439 genomic regions or 6,534 regions if overlapping predictions are combined.

The accuracy of the predictions was evaluated using a number of different annotations of human ncRNAs. Most of the ncRNAs used (214 miRNAs, 17 miscellaneous RNAs (Xist, Telomerase RNA, HVG-1,2 and 3, H19, RNase MRP, RNase P), 636 tRNAs, 705 rRNAs, 1805 small cytoplasmic RNAs (SRP, hY1, hY3, hY4, hY5) and 1103 snoRNAs) were mapped onto the human genome by Jones & Eddy [20]. In addition, we used the following ncRNA annotations: the ENSEMBL v37 ncRNA track, which annotates 4156 human ncRNAs [21], a set of 332 miRNAs obtained from miRBase (ver 8.0) [22], 1435 snoRNAs from snoRNA-LBME-db [23] and 441 tRNA and 170 Pseudo-tRNAs obtained from the genomic tRNA database [24].

Some predicted ncRNAs were also noted but these were not used for evaluating the accuracy of the predictions. These were 674, 133 and 975 miRNA candidates from miRMAP [33], the colorectal miRNAome [42] and miRNA shadowing [35] respectively. Overlaps with protein coding features were determined using the Refseq database [30].

### Cell culture

SK-N-AS neuroblastoma cells (ATCC # CRL-2137) were cultured as mono-layers in Dulbecco's modified eagle medium (Invitrogen) supplemented with 2 mM L-glutamate (Invitrogen), 10% bovine fetal serum (Invitrogen) and antibiotics (penicillin 50 units/ml and streptomycin and 50 μg/ml, Invitrogen) at 37°C and 5% CO2. Cells for RNA extraction were harvested at passages 8–20 at 90–95% confluence.

### Northern Blotting for small RNAs

RNA samples enriched for small RNAs were extracted using the mirVana extraction kit according to the recommendations of the manufacturer (Ambion). The integrity and concentration of the RNA samples was evaluated by spectrophotometry (Nano-drop ND-1000) and agarose gel electrophoresis.

2 μg of the small-selected RNA samples were run on 12% denaturing polyacrylamide gels together with the Decade marker (Ambion) for about 3 hours at 250 V. The gels were stained with ethidium bromide in 0,5 × TBE for 45 min. The RNA was blotted onto Hybond+ N membranes (Amersham Biosciences) in a semidry blotter (BIO-RAD trans-blot SD) at 20 V for 1 hour and crosslinked twice with auto crosslinking settings in a UV Stratalinker 1800 from Stratagene. Crosslinked membranes were stored at 4°C.

20 pmol of LNA modified DNA oligos (Sigma-Proligo) were end-labeled with α-^32^P UTP (3000 Ci/mmol, 10 mCi/ml, Amersham) using T4 PNK (Roche) and purified through NucAway spin columns according to the recommendations of the manufacturer (Ambion). 2–5 μl (of 20 μl total) of the eluates from the NucAway columns was added to 10 ml of Ultrahyb-Oligo hybridization buffer (Ambion) in hybridization tubes and used for hybridization of the blotted membranes over night at 42°C in an Apollo HP9300 hybridization oven. The blotted membranes was washed twice at 68°C for 30 min in wash buffer (2× SSC and 0,5% SDS). Films (Kodak) were exposed to the blotted membranes 2–6 days at -80°C using intensifying screens (Amersham). All northern blots were replicated at least twice with independent RNA preparations.

## Abbreviations

CRUFTS: Conserved RNAs of Unidentified Function that are Transcribed and Structured, ncRNA: noncoding RNA.

## Authors' contributions

CW performed and designed the northern blot experiments with help from JV and MHH. PPG did the bioinformatic analysis. JV wrote the manuscript with help from CW, PPG and MMH. PPG and JV conceived and designed the study.

## Supplementary Material

Additional file 1 alignments and structures of the experimentally investigated CRUFTS and the sequence of the probes used for northern blotting. Click here for file

Additional file 2 UCSC screenshots of the genomic neighborhoods of the verified covariance CRUFTS. Click here for file

Additional file 3 UCSC screenshots of the genomic neighborhoods of the verified hairpin CRUFTS. Click here for file

Additional file 4 Northern blots for the C2780 and C4801 CRUFTS on RNA isolated from the SK-N-AS, U87, U373, HeLa, C2C12, HUH-7 and MCF-7 cell lines. Click here for file
