# Supplementary material for: Use of tiling array data and RNA secondary structure predictions to identify noncoding RNA genes
Source: BMC Genomics. 2007 Jul 23;8:244. doi: 10.1186/1471-2164-8-244 (PMC1949828; doi:10.1186/1471-2164-8-244)
Supplement: Additional file 1 — alignments and structures of the experimentally investigated CRUFTS and the sequence of the probes used for northern blotting. [file 1471-2164-8-244-S1.pdf]

A

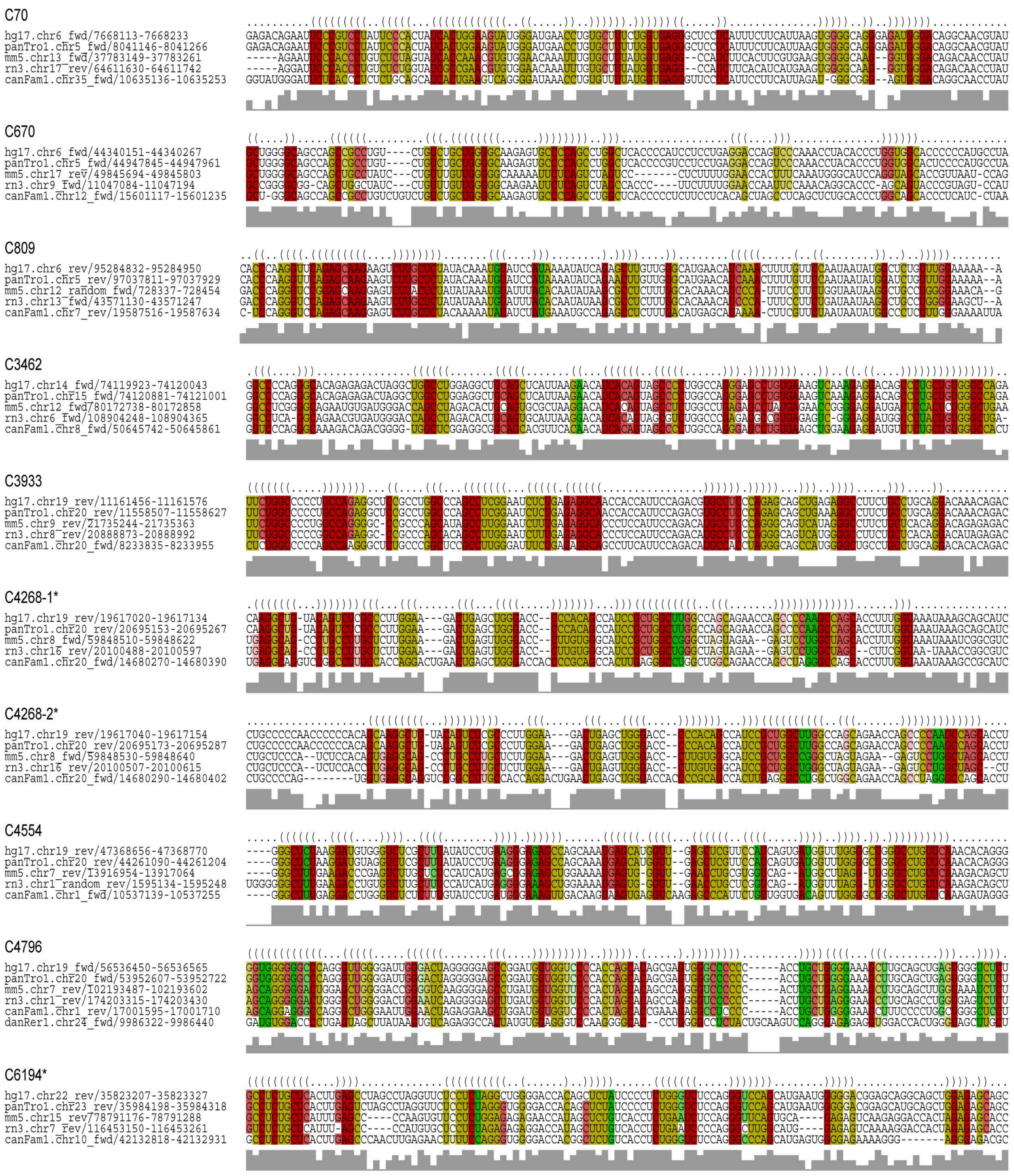

B

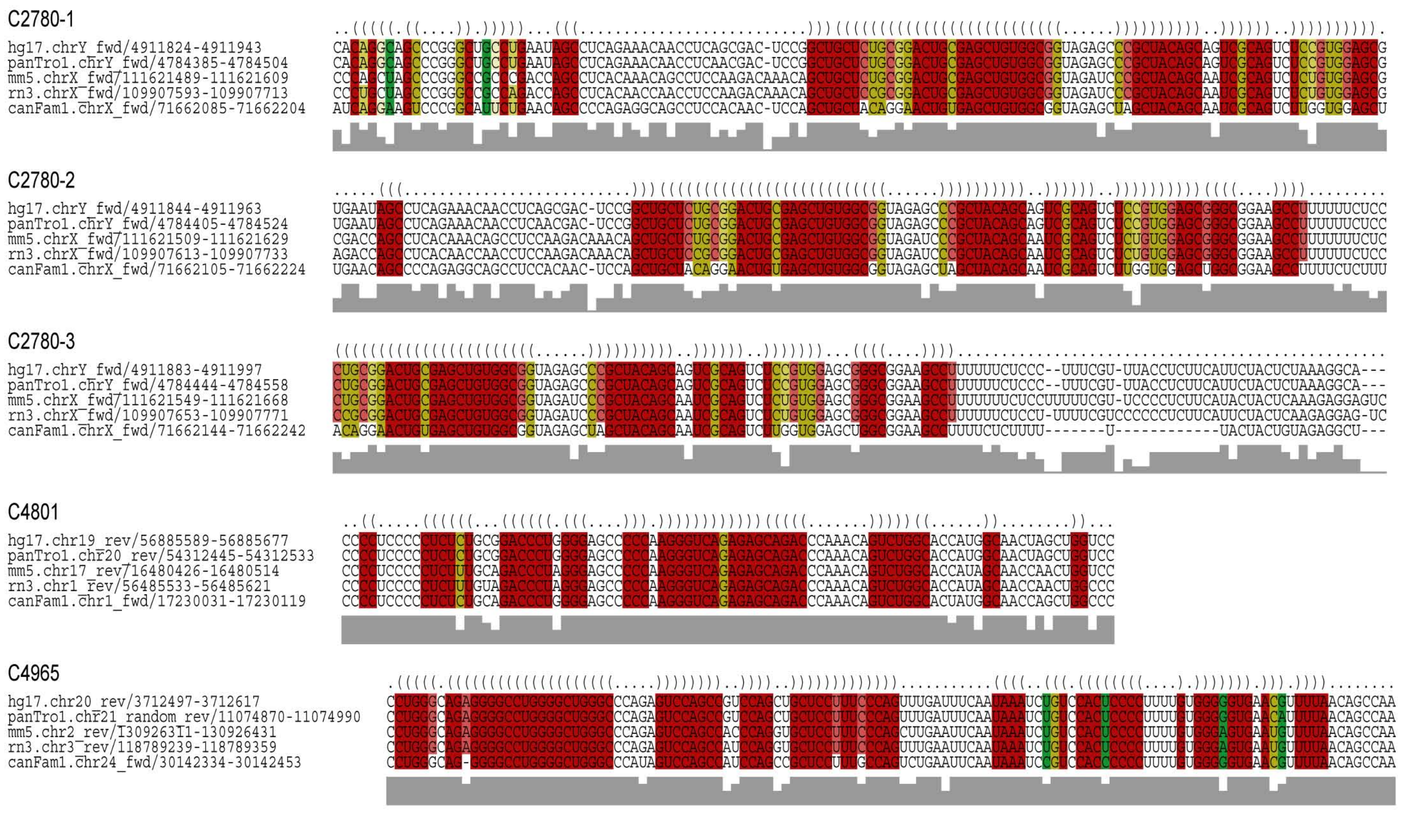

C

NAME	SEQUENCE	GENOMIC LOCATION		
C70	5´- cat ccc ata ctt cca gtg ata g - 3´	chr 6	+	7668140 - 7668161
C670	5´- cac tct tgc ccc agc aga cag a - 3´	chr 6	+	44340173 - 44340194
C809	5´- agc tct gat att tta tgg ata c - 3´	chr 6	-	95284889 - 95284910
C3462	5´- cct ctt tga ctt tca cag gct c - 3´	chr 14	+	74120001 - 74120022
C3933	5´- tgc agg cag aag gcc tct cag c - 3´	chr 19	-	11161469 - 11161490
C4268	5´- gag gca ggg ctt ttt aaa atc t - 3´	chr 19	-	19616997 - 19617018
C4554	5´- acc atc act gat gga acg agc t - 3´	chr 19	-	47368685 - 47368706
C4796	5´- ctc ccc cta gtc cca atc ccc a - 3´	chr 19	+	56536468 - 56536489
C6194	5´- ggg gcc agc cat aat taa gga c - 3´	chr 22	-	35823164 - 35823185
C2780-1	5´- cca cag ctc gca gtc cgc aga g - 3´	chr Y	+	4911882 - 4911903
C2780-2	5´- gga gac tgc gac tgc tgt agc t - 3´	chr Y	+	4911915 - 4911935
C4801-1	5´- gct ccc cag ggt ccg cag aga g - 3´	chr 19	-	56885647 - 56885668
C4801-2	5´- tct ctg acc ctt ggg ggc tcc c - 3´	chr 19	-	56885631 - 56885652
C4965-1	5´- gcc cag ccc cag gcc cct ctg c - 3´	chr 20	-	3712591 - 3712612
C4965-2	5´- aag gag cag ctg gac ggc tgg a - 3´	chr 20	-	3712563 - 3712584

Additional data-file 1: The CRUFTS and the probes used for their detection. A + B: Alignments, predicted secondary structures and conservation of the CRUFTS.
Alignments are 5-6 way of human (hg17), chimp (panTro1), mouse (mm5), rat (rn3), zebrafish (danRer1) and dog (canFam1). The chromosomal location, coordinates and the orientation of the CRUFTS are also listed. All sequences are in their 5' to 3' orientation.
The predicted consensus secondary structures are shown with parantheses and the corresponding positions in the alignments are color-coded according to the conservation of the interaction. Green indicates that 3 different types of pairs (e.g. G-C in human, G-U in dog and A-U in zebrafish) support the interaction. Yellow that it is supported by 2 types of pairs and red that only a single pair-type supports the interaction. The intensity of the colors fates with the number of sequences that conflicts with the predicted interaction.
The sequence-conservation is denoted with grey bars below the alignments with bar-height increasing with conservation.
C: The probes used for the northern blotting experiments. The probes are named according to the CRUFTS they were designed to detect. For each probe, its 5' to 3' sequence and the genomic coordinates (chromosome, orientation and position) of its match are listed. The genomic coordinates refers to the hg17 version of the human genome.
