# Supplementary figures and images for: Use of tiling array data and RNA secondary structure predictions to identify noncoding RNA genes
Source: BMC Genomics. 2007 Jul 23;8:244. doi: 10.1186/1471-2164-8-244 (PMC1949828; doi:10.1186/1471-2164-8-244)

Genomic neighborhoods of the verified covariance CRUFTS

C4796

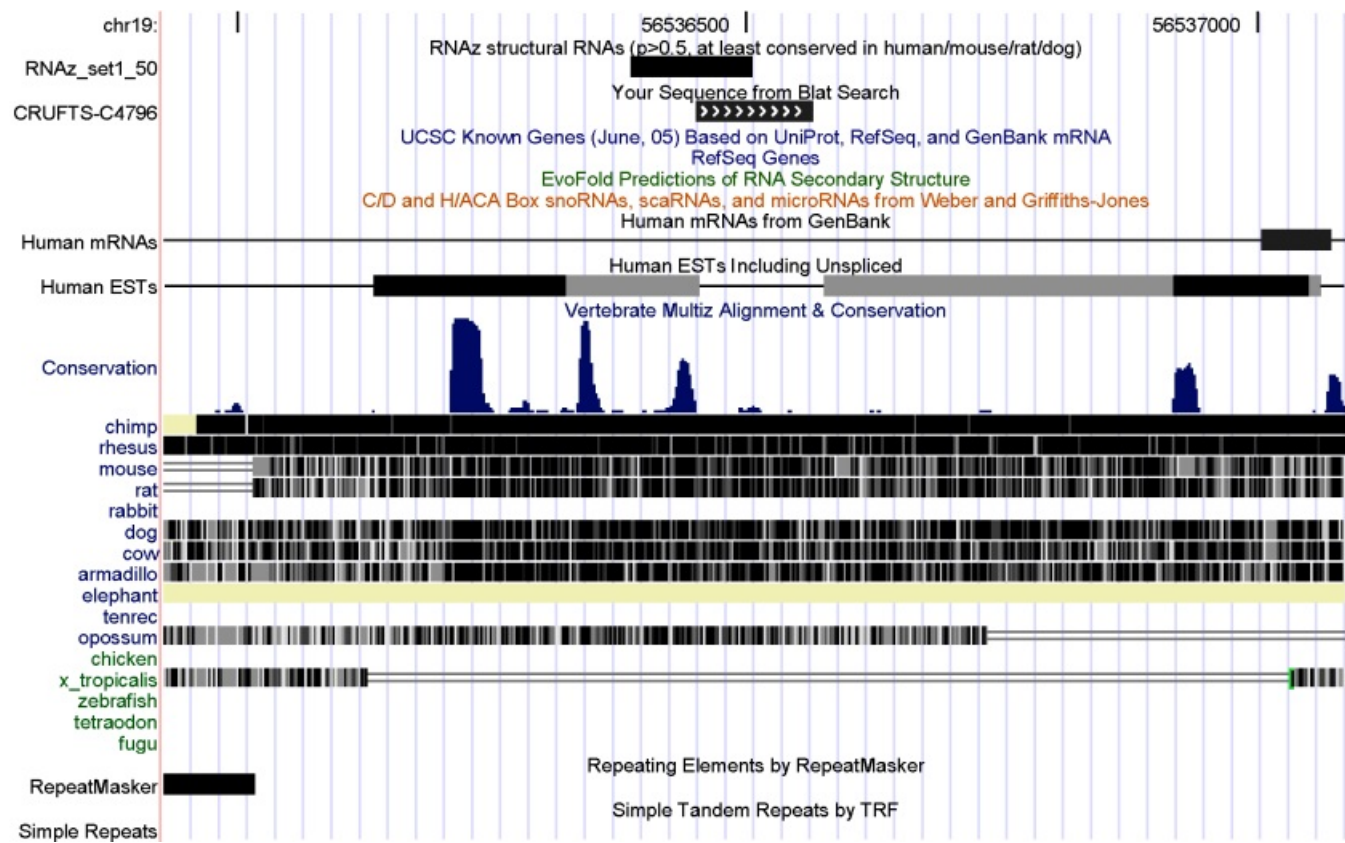

C6194

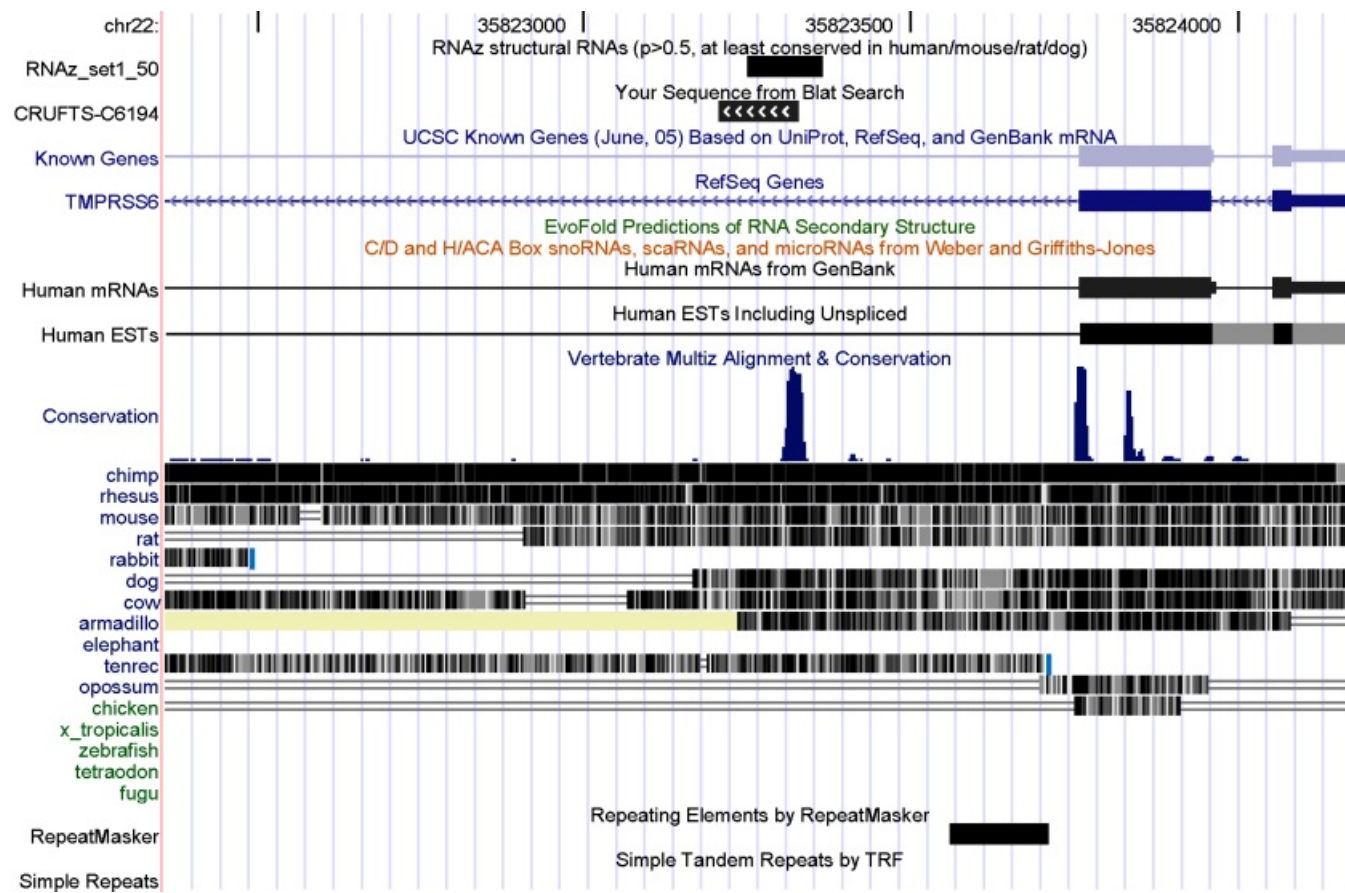

# C3462

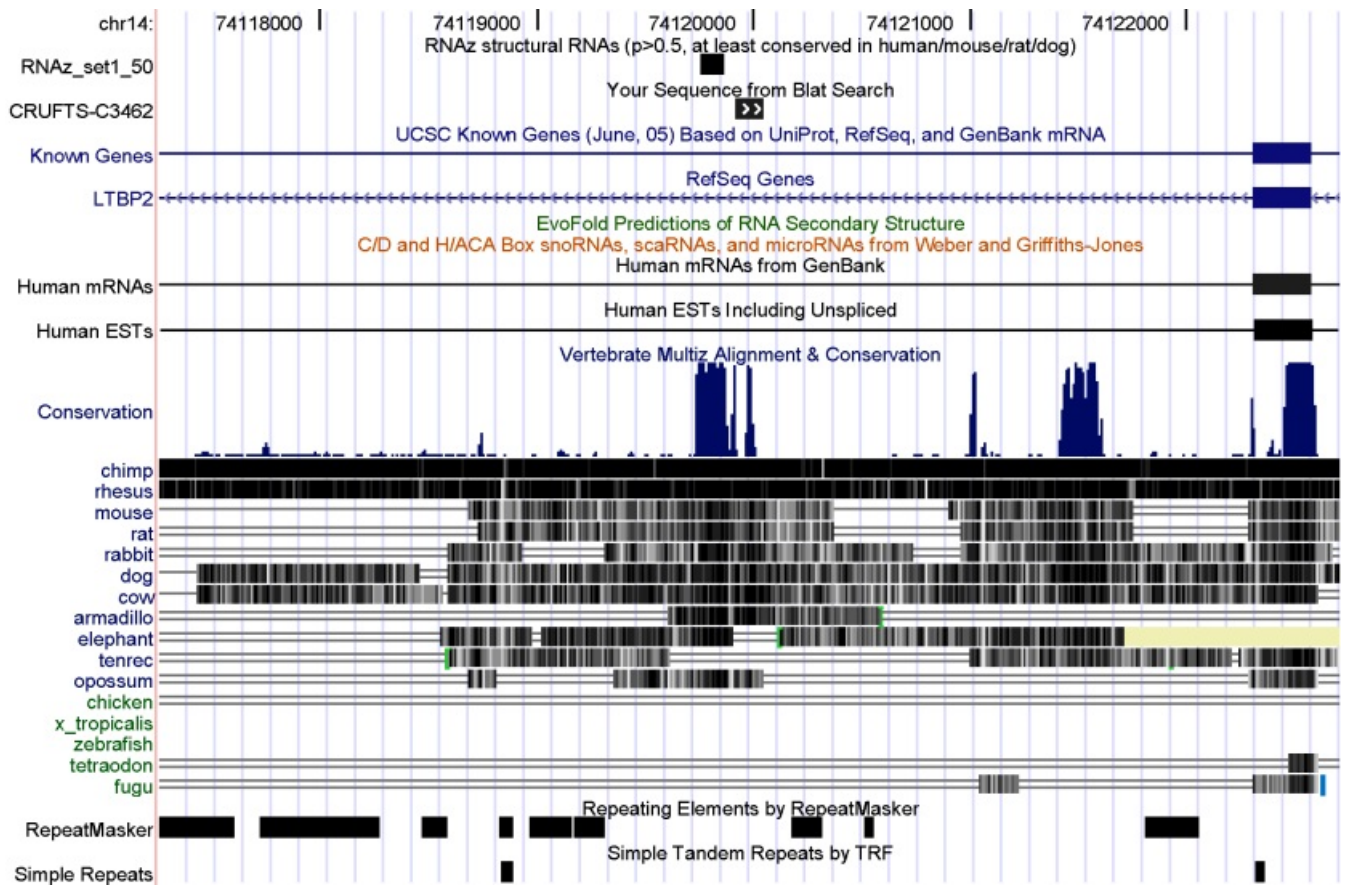

Supplement: Additional file 2 — UCSC screenshots of the genomic neighborhoods of the verified covariance CRUFTS. [file 1471-2164-8-244-S2.pdf]

# Genomic neighborhoods of the verified hairpin CRUFTS

## C2780

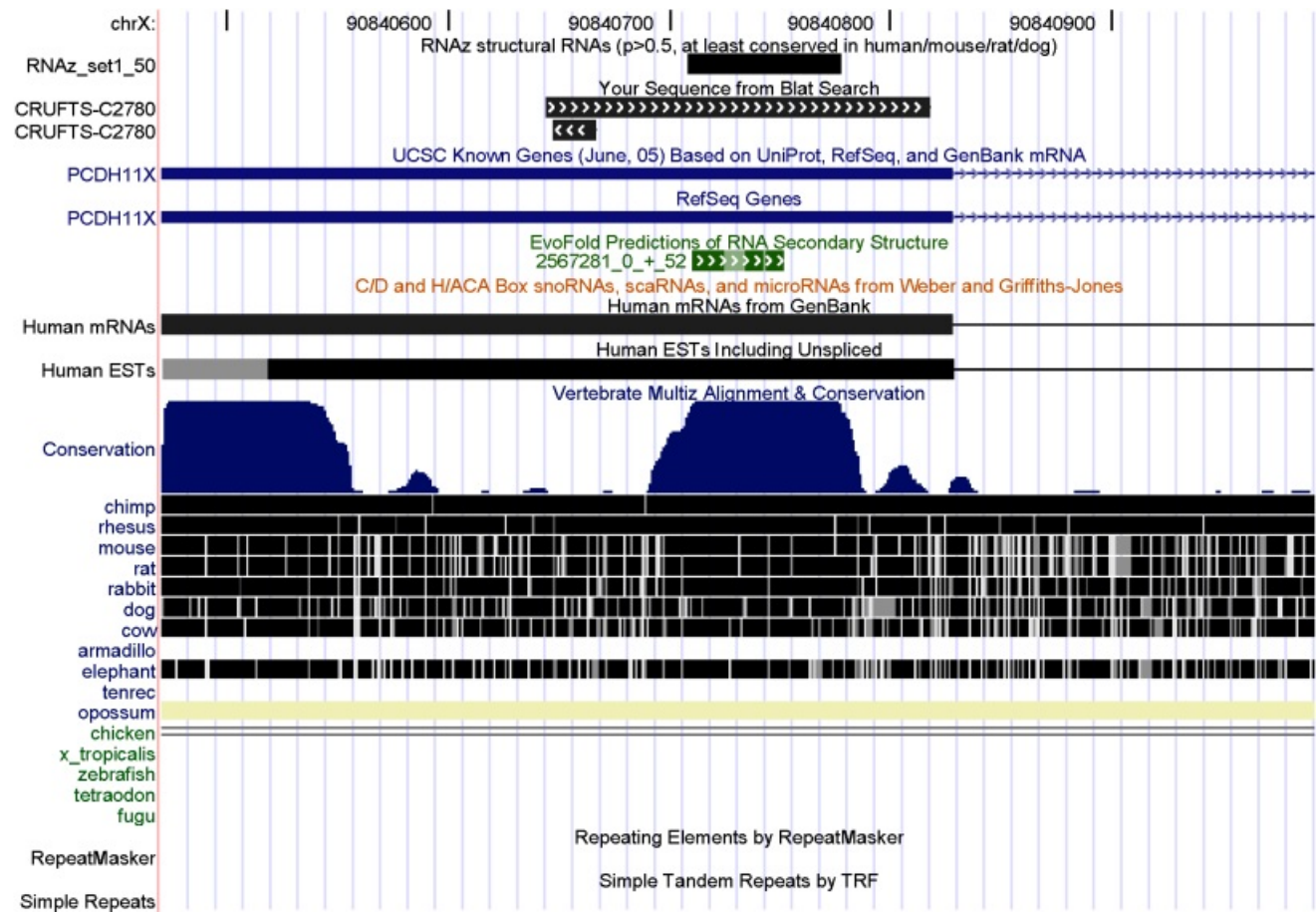

## C4801

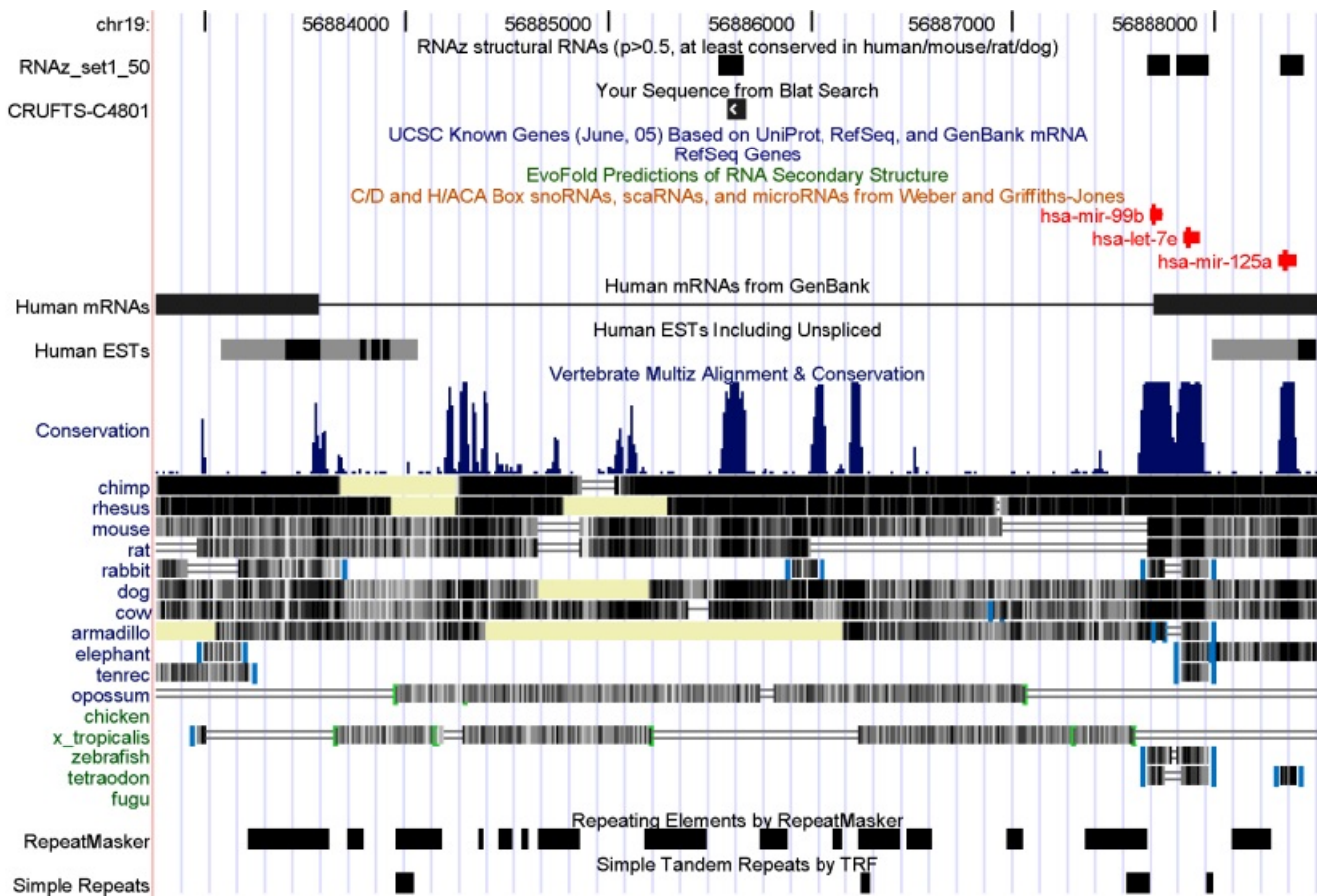

Supplement: Additional file 3 — UCSC screenshots of the genomic neighborhoods of the verified hairpin CRUFTS. [file 1471-2164-8-244-S3.pdf]
