# Supplementary material for: Use of tiling array data and RNA secondary structure predictions to identify noncoding RNA genes
Source: BMC Genomics. 2007 Jul 23;8:244. doi: 10.1186/1471-2164-8-244 (PMC1949828; doi:10.1186/1471-2164-8-244)
Supplement: Additional file 4 — Northern blots for the C2780 and C4801 CRUFTS on RNA isolated from the SK-N-AS, U87, U373, HeLa, C2C12, HUH-7 and MCF-7 cell lines. [file 1471-2164-8-244-S4.pdf]

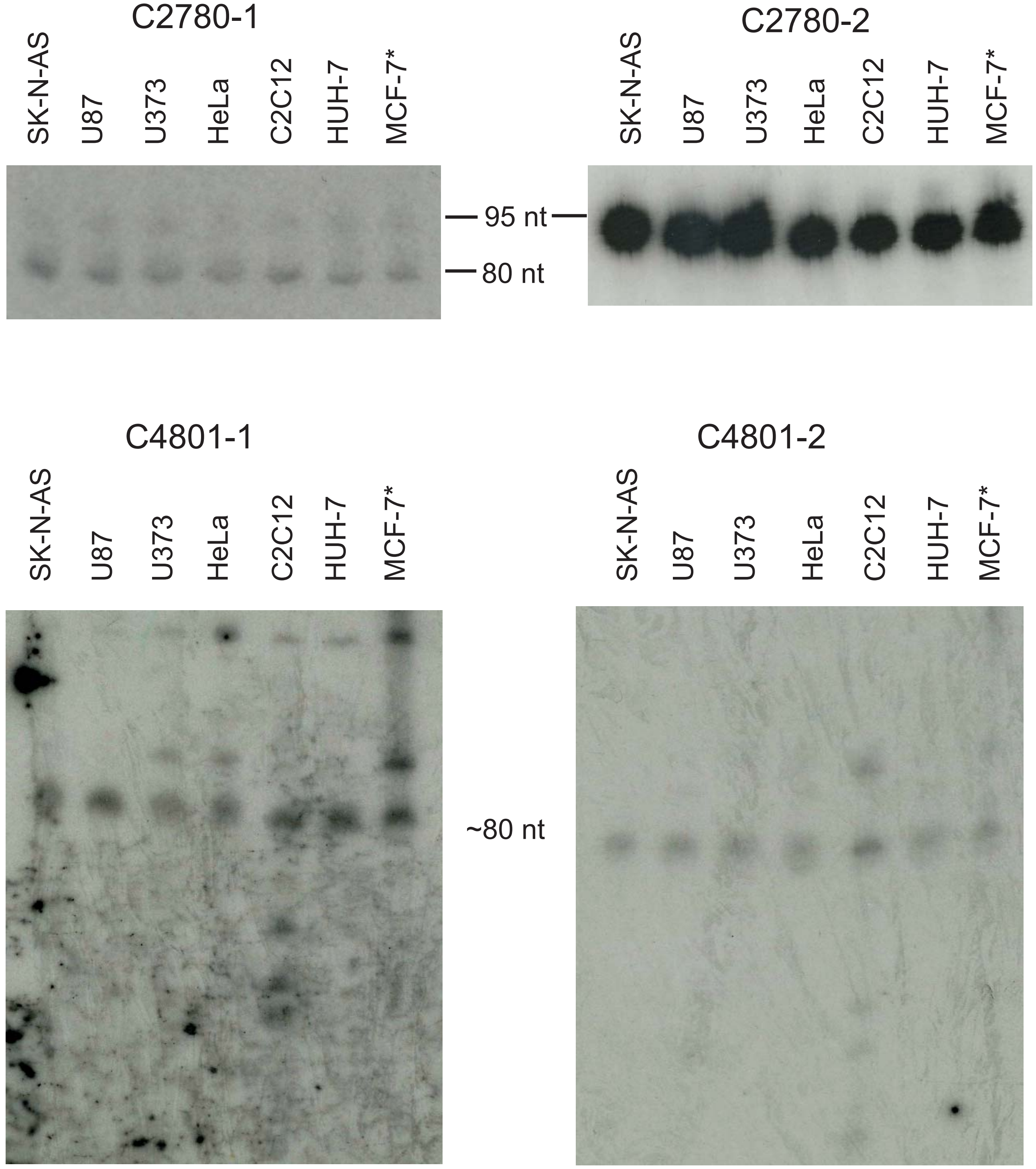

Additional file 4: Northern blots for CRUFTS C2780 and C4801 on RNA isolated from seven different cancer cell lines. The cell lines used were SK-N-AS (neuroblastoma), U87 (glia), U373 (glia), HeLa (Cervix), C2C12 (skeletal muscle),HUH-7 (liver) and MCF-7 (breast) cell lines. For MCF-7 total RNA was used. For all cell lines small-selected RNA samples were used (miRvana protocol, Ambion). No signal of mature miRNA size could be detected in any of the cell lines, although the probes for CRUFTS C4801 did give additional signals in cell line C2C12.
